# Supplementary material for: Development and validation of an instrument to measure personality in adolescence: The HEXACO Medium School Inventory Extended (MSI-E)
Source: PLoS One. 2023 Jan 20;18(1):e0280563. doi: 10.1371/journal.pone.0280563 (PMC9858823; doi:10.1371/journal.pone.0280563)
Supplement: S1 File — (DOCX) [file pone.0280563.s001.docx]

**S1 File. Supplementary materials**

**Table A.**

*Factor loadings (oblimin rotation) of the Principal Component Analysis on the items of the 190-item HEXACO-MSI-E (Study 1).*

| **Item** | **Reverse** | **Stem** | **H** | **E** | **X** | **A** | **C** | **O** |
| --- | --- | --- | --- | --- | --- | --- | --- | --- |
| H.Fair.1 |  | I would never take things that aren't mine | **.458** | .024 | -.090 | -.009 | .234 | .018 |
| H.Fair.10 |  | I would never steal something from another guy/girl | **.456** | .060 | -.174 | -.011 | .148 | .127 |
| H.Fair.11 | R | I'd like to find a way to get things from the stores without paying | **-.405** | .062 | .108 | .009 | -.111 | -.077 |
| H.Fair.12 | R | I cheat if I'm sure I won't get caught | **-.453** | .054 | .121 | .030 | -.185 | .122 |
| H.Fair.14 | R | I'm curious how I can make a lot of money dishonestly | **-.482** | .145 | .136 | -.181 | -.051 | .014 |
| H.Fair.3 |  | If a cashier accidentally gives me extra change, I return it | **.360** | -.060 | -.063 | -.018 | .222 | -.078 |
| H.Fair.8 | R | If I happen to take advantage of someone, I don't regret it | **-.413** | .077 | .078 | .000 | .002 | .008 |
| H.Fair.9 |  | I wouldn't steal even if I was sure I'd get away with it | **.472** | -.005 | -.123 | -.054 | .150 | .051 |
| H.Gree.1 |  | I wouldn't like being a famous celebrity | **.328** | .114 | .199 | -.155 | .052 | -.015 |
| H.Gree.11 |  | It's not important to have a lot of money | **.435** | -.003 | -.012 | -.160 | -.072 | -.038 |
| H.Gree.12 | R | I would like friends who have expensive and fashionable items (mobile, clothes, games) | **-.495** | .017 | .005 | -.056 | .046 | .088 |
| H.Gree.13 | R | I really like to have expensive and branded items (mobile phone, clothes, games) | **-.453** | .012 | -.137 | .133 | .073 | .114 |
| H.Gree.14 | R | I want others to see how important I am | **-.406** | -.102 | -.101 | .131 | .047 | -.032 |
| H.Gree.16 | R | I want to become famous | **-.410** | -.113 | -.245 | .083 | -.056 | -.053 |
| H.Gree.3 |  | I'm not attracted to luxury | **.449** | .062 | .045 | -.204 | -.106 | -.105 |
| H.Gree.4 |  | I'm not interested in power | **.440** | .076 | .044 | -.200 | -.103 | -.008 |
| H.Mode.1 |  | I don't think I'm better than other people | **.463** | -.071 | .067 | -.157 | -.115 | .032 |
| H.Mode.10 | R | People have to behave better with me than with the others | **-.417** | .102 | .036 | .020 | .035 | .025 |
| H.Mode.11 | R | Some say I give myself a lot of airs | **-.369** | .061 | -.003 | .159 | .029 | -.060 |
| H.Mode.16 | R | I'm entitled to special treatment | **-.444** | .037 | -.031 | -.007 | .121 | .049 |
| H.Mode.3 |  | I don't think I'm any better than the others | **.390** | -.046 | .112 | -.142 | -.115 | -.012 |
| H.Mode.4 |  | I don't like to attract attention | **.416** | .061 | .205 | -.112 | .106 | .065 |
| H.Mode.6 | R | I'll show off if I get the chance | **-.439** | .031 | -.224 | .047 | -.025 | -.013 |
| H.Mode.7 |  | I'm not the type to brag about my virtues | **.448** | -.002 | .005 | -.070 | -.119 | -.001 |
| H.Sinc.1 |  | I don't pretend to be better than I am | **.319** | .056 | .000 | -.070 | .060 | .027 |
| H.Sinc.10 | R | If I want to get something from people, I try to make friends with them | **-.489** | .012 | .059 | -.032 | -.001 | .011 |
| H.Sinc.11 | R | I say nice things to my teachers to get higher grades | **-.451** | .042 | .023 | -.178 | -.065 | .099 |
| H.Sinc.13 | R | Sometimes I pretend to be better than I really am | **-.382** | -.066 | .192 | .036 | -.046 | .031 |
| H.Sinc.14 | R | Sometimes I lie to get what I want | **-.467** | -.027 | .005 | .085 | -.248 | .105 |
| H.Sinc.15 |  | I'm not good at pretending with other people | **.386** | -.087 | -.041 | -.110 | .118 | .126 |
| H.Sinc.16 |  | I find it difficult to lie | **.368** | -.027 | -.015 | -.231 | .192 | .026 |
| H.Sinc.4 |  | I'm not good at acting to impress people | **.415** | -.098 | .046 | .049 | .013 | .168 |
| E.Anxi.11 | R | I worry about small things | .027 | **-.348** | .166 | .041 | .144 | -.271 |
| E.Anxi.12 |  | I worry less than other people | -.138 | **.347** | .058 | .046 | -.081 | .121 |
| E.Anxi.14 | R | I often worry that something may go wrong | .075 | **-.298** | .063 | .067 | .151 | -.116 |
| E.Anxi.15 |  | I do not worry as much as other people | .007 | **.366** | -.053 | .052 | -.140 | .163 |
| E.Anxi.2 | R | I worry about everything | .099 | **-.338** | .171 | .056 | .140 | -.116 |
| E.Anxi.5 | R | I am easily panicked | .059 | **-.496** | .224 | .102 | .015 | .079 |
| E.Anxi.8 |  | I am not easily upset by what happens around me | -.009 | **.282** | -.078 | -.127 | .030 | .014 |
| E.Anxi.9 |  | I remain calm under pressure | .082 | **.428** | -.057 | -.245 | .078 | -.010 |
| E.Depe.1 | R | I need reassurance | .033 | **-.443** | .164 | .021 | -.079 | -.016 |
| E.Depe.11 |  | I can solve difficult situations without encouragement | .039 | **.508** | -.022 | .040 | .050 | -.128 |
| E.Depe.13 |  | I can face all my personal problems on my own | .039 | **.488** | .146 | .174 | .087 | -.143 |
| E.Depe.14 |  | I rarely need other people's support | .061 | **.380** | .049 | .087 | .039 | .100 |
| E.Depe.15 | R | I need other people's comfort | -.067 | **-.512** | .053 | -.035 | -.073 | -.096 |
| E.Depe.16 |  | I can easily overcome difficulties by myself | .038 | **.533** | .010 | .155 | .114 | -.162 |
| E.Depe.7 | R | I seek support | -.053 | **-.419** | .113 | -.041 | -.128 | -.052 |
| E.Depe.9 | R | When something bad happens, I need someone to cheer me up | -.044 | **-.543** | -.172 | -.157 | -.030 | -.018 |
| E.Fear.11 | R | If there is a danger, I am very much afraid | .102 | **-.492** | .035 | .061 | .180 | .234 |
| E.Fear.14 |  | I can easily bear physical pain | .044 | **.412** | -.047 | -.109 | -.149 | -.112 |
| E.Fear.15 | R | I am afraid of feeling pain | .027 | **-.531** | -.021 | .045 | .111 | .158 |
| E.Fear.16 |  | I am braver than other people in dangerous situations | -.185 | **.452** | -.080 | -.038 | -.192 | -.242 |
| E.Fear.6 | R | I am afraid of travelling when the weather is bad | -.029 | **-.272** | .023 | .006 | .142 | .104 |
| E.Fear.7 |  | Even when I get hurt, I resist and try not to cry | .016 | **.380** | -.019 | -.087 | .004 | -.028 |
| E.Fear.8 |  | I play dangerous games fearlessly | -.199 | **.364** | .015 | .010 | -.281 | -.182 |
| E.Fear.9 |  | They tell me I am afraid of nothing | -.120 | **.454** | -.073 | -.087 | -.051 | -.208 |
| E.Sent.10 | R | If other people cry, I feel like crying too | .036 | **-.414** | -.017 | -.052 | -.059 | -.199 |
| E.Sent.13 | R | When somebody is in pain, I feel pain too | .081 | **-.357** | -.031 | -.102 | -.038 | -.186 |
| E.Sent.14 | R | Sometimes I feel like crying, when I say someone goodbye | .147 | **-.399** | -.081 | -.019 | -.070 | -.146 |
| E.Sent.2 | R | I immediately feel sad when I hear of an unhappy event | -.005 | **-.372** | -.027 | -.114 | .049 | -.094 |
| E.Sent.3 | R | I can cry during a film | -.085 | **-.380** | .125 | .006 | -.008 | -.284 |
| E.Sent.4 |  | I am scarcely sensitive to other people’s needs | -.118 | **.307** | .130 | .051 | .098 | .167 |
| E.Sent.8 |  | I can’t understand emotional people | -.035 | **.364** | .171 | .063 | .066 | .161 |
| E.Sent.9 |  | I am rarely moved | -.012 | **.361** | .181 | .007 | .181 | .186 |
| X.Live.10 | R | I'm usually cheerful and positive | .111 | -.043 | **-.578** | -.176 | .056 | .047 |
| X.Live.13 |  | Most of my friends are more optimistic and active than I am | .057 | .018 | **.466** | -.009 | -.077 | .050 |
| X.Live.14 |  | I'm often in a gloomy mood | -.150 | -.018 | **.507** | .071 | -.121 | -.065 |
| X.Live.15 | R | I'm usually cheerful | .140 | -.024 | **-.564** | -.101 | .046 | .070 |
| X.Live.3 | R | I smile so much | .012 | -.047 | **-.509** | -.142 | .015 | -.048 |
| X.Live.6 |  | I don't think I have much inner strength | .022 | -.248 | **.429** | -.067 | -.043 | .129 |
| X.Live.7 | R | I have so much fun | .043 | .046 | **-.572** | -.089 | -.062 | .052 |
| X.Live.9 |  | I often feel down | -.028 | -.178 | **.463** | .143 | -.142 | -.114 |
| X.Socb.10 | R | At parties, I'm often the one who approaches others to talk | -.068 | .035 | **-.282** | -.169 | -.087 | -.140 |
| X.Socb.11 |  | When I'm in a group, I only speak when someone addresses me directly | .053 | -.038 | **.459** | -.063 | .087 | .072 |
| X.Socb.13 |  | I stay in the background when I'm in a group | .076 | -.119 | **.551** | -.115 | .040 | .001 |
| X.Socb.4 | R | I have a strong personality | -.106 | .199 | **-.344** | .092 | .048 | -.171 |
| X.Socb.5 |  | I'd be afraid to speak in public | .206 | -.223 | **.346** | -.013 | -.016 | .236 |
| X.Socb.6 |  | I find it difficult to approach others | .087 | .022 | **.530** | .059 | .061 | .082 |
| X.Socb.8 |  | I have little to say | .124 | .118 | **.386** | -.109 | .034 | .245 |
| X.Socb.9 |  | When there are a lot of people I can't say what I think | .067 | -.257 | **.317** | -.106 | -.040 | .181 |
| X.Soci.1 | R | I usually like to spend my free time with people | .040 | -.102 | **-.527** | -.124 | .052 | .085 |
| X.Soci.10 | R | It's nice to have a lot of friends to talk to | .181 | -.144 | **-.546** | -.003 | .009 | .136 |
| X.Soci.12 | R | When I'm in a new place, I try to make friends immediately | -.102 | -.048 | **-.539** | -.165 | -.032 | -.076 |
| X.Soci.14 |  | I prefer to be alone | .091 | .259 | **.549** | .122 | .016 | -.100 |
| X.Soci.3 | R | I make friends easily | -.084 | -.006 | **-.582** | -.079 | -.096 | -.078 |
| X.Soci.5 |  | I seem to get less pleasure than others from interacting with people | -.069 | .037 | **.548** | .001 | -.022 | .049 |
| X.Soci.6 |  | I rarely enjoy being with people | -.147 | .111 | **.390** | -.097 | .092 | .053 |
| X.Soci.7 |  | I keep others at a distance | -.166 | .120 | **.383** | .068 | .072 | -.095 |
| X.SSes.10 | R | I'm pleased with myself | .093 | .192 | **-.543** | .017 | .148 | .103 |
| X.SSes.13 | R | Others enjoy spending time with me | .090 | .000 | **-.586** | .045 | .113 | .029 |
| X.SSes.2 |  | I don't think the others like me very much | .039 | -.181 | **.537** | .015 | -.143 | -.067 |
| X.SSes.3 |  | I feel unimportant | .085 | -.173 | **.568** | .009 | -.135 | -.112 |
| X.SSes.4 | R | People like me | -.024 | .065 | **-.569** | -.029 | .138 | .008 |
| X.SSes.6 |  | Nobody likes talking to me | .048 | -.050 | **.522** | -.059 | -.097 | -.111 |
| X.SSes.8 |  | I don't have much self-esteem | .042 | -.234 | **.543** | .039 | -.131 | -.056 |
| X.SSes.9 | R | I hardly ever feel ignored | .064 | .179 | **-.506** | -.092 | .063 | .180 |
| A.Flex.10 |  | My parents say I always want to be right | -.047 | -.047 | -.055 | **.491** | -.118 | -.009 |
| A.Flex.11 |  | When people tell me I'm wrong, I get in arguments with them | -.241 | .038 | .138 | **.342** | -.066 | .020 |
| A.Flex.12 |  | When I think I'm right, I don't change my mind one iota | .017 | .017 | -.131 | **.425** | -.048 | -.029 |
| A.Flex.15 |  | Others struggle to change my mind | .043 | .066 | -.035 | **.470** | -.071 | -.101 |
| A.Flex.2 | R | I am good at taking advice | .001 | -.089 | -.126 | **-.363** | .187 | -.023 |
| A.Flex.4 |  | I get angry if other people change the way I set things up | -.141 | -.058 | .009 | **.378** | .059 | .027 |
| A.Flex.5 |  | I hate to be contradicted | -.144 | -.014 | .030 | **.343** | -.081 | .034 |
| A.Flex.6 |  | I am hard to please | -.070 | .080 | .067 | **.367** | -.093 | .018 |
| A.Forg.10 | R | If someone makes me angry, I usually get over it soon | .173 | -.016 | -.107 | **-.517** | -.068 | .025 |
| A.Forg.11 | R | If people treat me badly, I forgive them and forget about it | .113 | -.046 | -.043 | **-.543** | -.061 | -.038 |
| A.Forg.12 |  | If someone misbehaves with me, they can't be my friend anymore | -.110 | .099 | .146 | **.342** | .070 | .058 |
| A.Forg.13 |  | I remain unfriendly to those who have been bad to me | .011 | .071 | .182 | **.523** | .128 | .122 |
| A.Forg.14 | R | I'm quick to restore confidence to those who tricked and cheated me | -.009 | -.060 | .039 | **-.480** | -.098 | -.061 |
| A.Forg.15 |  | I'm wary of people who have wronged me | -.002 | .037 | -.043 | **.375** | .043 | .060 |
| A.Forg.2 | R | I'm prone to forgiving others | .143 | -.191 | -.114 | **-.458** | -.077 | -.024 |
| A.Forg.4 |  | I find it hard to forgive others | .012 | .165 | .108 | **.498** | .117 | .088 |
| A.Gent.1 | R | I rarely complain | .052 | .152 | -.013 | **-.391** | .110 | .028 |
| A.Gent.12 | R | Even when people make many mistakes, I don't judge them negatively | .298 | .028 | -.084 | **-.319** | -.040 | -.049 |
| A.Gent.13 |  | I often express criticism | -.185 | -.035 | .043 | **.315** | -.082 | -.044 |
| A.Gent.15 |  | I react negatively to people who make mistakes | -.165 | .010 | .022 | **.316** | .078 | .116 |
| A.Gent.16 |  | You can see right away if something seems stupid to me | -.032 | .019 | -.095 | **.252** | .094 | .083 |
| A.Gent.5 |  | I judge others very quickly | -.139 | .032 | .037 | **.280** | -.091 | .150 |
| A.Gent.6 |  | I find flaws in everything | -.142 | -.077 | .195 | **.314** | -.128 | .067 |
| A.Gent.9 | R | I'm a nice, quiet guy/girl | .271 | -.137 | -.149 | **-.294** | .221 | .046 |
| A.Pati.1 | R | It takes a lot to make me angry at someone | .050 | .029 | -.083 | **-.520** | .102 | -.007 |
| A.Pati.11 |  | My parents think I get angry easily | .047 | -.053 | .107 | **.532** | -.152 | .019 |
| A.Pati.15 | R | Even when I'm treated badly, I stay calm | .101 | .092 | .079 | **-.502** | .060 | -.076 |
| A.Pati.3 | R | I'm usually a patient person | .031 | -.010 | .000 | **-.464** | .212 | -.077 |
| A.Pati.5 | R | I rarely get angry | -.059 | .123 | -.040 | **-.502** | .077 | .091 |
| A.Pati.6 |  | I'm easily irritable | .068 | -.033 | .115 | **.593** | -.162 | -.068 |
| A.Pati.7 |  | I get angry easily | .039 | -.026 | .130 | **.573** | -.145 | .052 |
| A.Pati.9 |  | I lose my patience | -.024 | -.102 | .084 | **.571** | -.162 | .030 |
| C.Dili.1 |  | I start right away when I have work to do | .049 | .142 | -.100 | -.114 | **.455** | -.139 |
| C.Dili.13 |  | I try harder than anyone else | -.121 | .051 | -.102 | .112 | **.379** | -.198 |
| C.Dili.15 | R | I postpone as long as possible the tasks that seem difficult to me | -.156 | -.083 | .054 | -.050 | **-.362** | .225 |
| C.Dili.2 |  | I'm very demanding when I do my homework | .116 | .059 | -.112 | .050 | **.469** | -.200 |
| C.Dili.3 |  | I work hard | .200 | -.008 | -.135 | .071 | **.515** | -.173 |
| C.Dili.4 |  | I finish my tasks successfully | .279 | .052 | -.162 | .178 | **.426** | -.199 |
| C.Dili.5 | R | I only do my tasks to get by | -.165 | .113 | .164 | -.120 | **-.341** | .180 |
| C.Dili.7 |  | I study a lot | .297 | -.049 | -.098 | .140 | **.455** | -.299 |
| C.Orga.1 |  | I keep things in order | -.107 | -.025 | .035 | -.159 | **.681** | .072 |
| C.Orga.10 |  | I like to keep things organized | -.007 | .000 | -.055 | -.022 | **.580** | .004 |
| C.Orga.11 |  | To avoid confusion, I decide what I have to do first | .089 | .006 | -.051 | -.032 | **.423** | -.033 |
| C.Orga.15 | R | I have trouble finding things because I'm messy | .130 | .006 | -.021 | .120 | **-.646** | -.095 |
| C.Orga.3 |  | I like to tidy up | -.121 | -.024 | .018 | -.164 | **.600** | -.001 |
| C.Orga.5 | R | My room is often messy | .145 | .014 | -.031 | .155 | **-.535** | -.132 |
| C.Orga.6 | R | I leave my things lying around | .198 | -.006 | -.104 | .178 | **-.600** | -.091 |
| C.Orga.8 | R | I often forget to put things back where they belong | .136 | .026 | -.045 | .159 | **-.522** | -.006 |
| C.Perf.10 |  | I always try to be precise in my homework, even if it takes me longer | .205 | -.085 | -.103 | .065 | **.510** | -.203 |
| C.Perf.11 |  | People consider me very precise | .023 | -.017 | -.110 | .128 | **.537** | -.146 |
| C.Perf.12 |  | When I write, I'm careful not to make mistakes | .192 | .017 | -.081 | .115 | **.440** | -.168 |
| C.Perf.13 |  | I usually check carefully how I did my homework | .173 | -.001 | -.085 | .033 | **.528** | -.261 |
| C.Perf.14 | R | I think it's a waste of time checking my homework for mistakes | -.209 | .083 | -.004 | -.083 | **-.354** | .192 |
| C.Perf.15 |  | I'm very precise when I do my homework | .211 | .046 | -.103 | .165 | **.585** | -.224 |
| C.Perf.2 |  | I keep on going until everything is perfect | -.106 | .043 | -.047 | .058 | **.475** | -.093 |
| C.Perf.9 | R | I'm not very precise when I do my homework | -.164 | .058 | .082 | -.201 | **-.540** | .181 |
| C.Prud.1 |  | I make plans and I stick to them | .009 | .046 | -.085 | -.094 | **.332** | -.189 |
| C.Prud.11 | R | I'm often wrong because I do things without thinking | -.129 | -.036 | .003 | .067 | **-.513** | .141 |
| C.Prud.13 |  | I think carefully before I do something dangerous | .163 | -.175 | -.057 | -.095 | **.440** | .093 |
| C.Prud.16 | R | I often do things without really thinking about it | -.166 | .035 | .103 | .063 | **-.494** | .001 |
| C.Prud.2 |  | I do things according to a plan | -.085 | .129 | .043 | -.077 | **.372** | -.055 |
| C.Prud.3 | R | I dive into things without thinking | -.032 | .071 | -.001 | .125 | **-.541** | -.003 |
| C.Prud.4 |  | When I do something I always think about the consequences | .043 | -.049 | .001 | -.205 | **.461** | .009 |
| C.Prud.5 | R | I make rash decisions | -.088 | .032 | -.026 | .118 | **-.403** | .075 |
| O.Aesa.1 | R | I believe in the importance of art | .091 | .000 | .012 | -.108 | .159 | **-.494** |
| O.Aesa.10 |  | I get bored visiting a museum | -.039 | .041 | -.110 | .112 | -.118 | **.499** |
| O.Aesa.11 |  | Reading poetry is a waste of time | -.196 | .069 | -.077 | .087 | -.197 | **.428** |
| O.Aesa.13 | R | I could be watching a painting for a long time | .034 | .006 | .219 | -.218 | .079 | **-.570** |
| O.Aesa.14 |  | I think most art is boring | -.100 | .031 | -.121 | .052 | -.116 | **.602** |
| O.Aesa.15 | R | I love poetry | .079 | -.062 | .104 | -.208 | .151 | **-.511** |
| O.Aesa.5 | R | I've read some classics of literature | .051 | .096 | .020 | -.022 | .161 | **-.352** |
| O.Aesa.6 |  | I don't like art | -.011 | .010 | -.112 | .062 | -.139 | **.484** |
| O.Crea.1 |  | I'm not a guy/girl with original thoughts and ideas | .108 | .046 | .147 | -.121 | -.006 | **.424** |
| O.Crea.10 | R | I'd like to create a work of art, such as a story, a song or a painting | .021 | -.055 | .041 | -.111 | .040 | **-.547** |
| O.Crea.11 | R | People tell me I have a lot of imagination | -.047 | -.076 | -.161 | -.014 | -.052 | **-.509** |
| O.Crea.12 |  | I'll never become an artist | .139 | .077 | .035 | .053 | .011 | **.413** |
| O.Crea.13 | R | I have a lot of imagination | -.054 | -.121 | -.210 | .021 | -.028 | **-.426** |
| O.Crea.14 | R | I like to think of new ways of doing things | -.042 | -.002 | -.146 | -.125 | .135 | **-.303** |
| O.Crea.3 |  | I don't have many ideas | .112 | .038 | .230 | -.133 | -.105 | **.488** |
| O.Crea.6 |  | I don't have a good imagination | .067 | .087 | .183 | -.093 | -.054 | **.398** |
| O.Inqu.1 | R | I'm interested in science | .064 | -.002 | -.052 | -.047 | .219 | **-.405** |
| O.Inqu.10 | R | I like to know what happens in other countries | .070 | .009 | .000 | -.024 | .193 | **-.375** |
| O.Inqu.11 |  | Documentaries about nature are boring | -.077 | .011 | -.059 | .131 | -.089 | **.399** |
| O.Inqu.14 | R | I like to read about new scientific discoveries | .025 | .027 | -.029 | -.065 | .157 | **-.505** |
| O.Inqu.15 |  | I think science is boring | -.043 | .024 | -.028 | .020 | -.159 | **.493** |
| O.Inqu.16 | R | I'd like to read a book about nature | .119 | .023 | .128 | -.193 | .167 | **-.460** |
| O.Inqu.7 |  | I avoid reading things that are too complicated | -.021 | -.104 | -.094 | -.014 | -.252 | **.400** |
| O.Inqu.8 |  | I'm not worried about political and social problems | .063 | .098 | -.038 | .109 | -.164 | **.301** |
| O.Unco.12 | R | I like people who have different ideas | .171 | .059 | -.021 | .085 | -.150 | **-.242** |
| O.Unco.13 |  | It's boring to talk about abstract things | .014 | .083 | -.108 | .067 | -.059 | **.416** |
| O.Unco.14 |  | It would bother me if people thought I was weird | -.032 | -.113 | -.193 | -.026 | .212 | **.344** |
| O.Unco.16 | R | People are surprised by my opinions | -.118 | .136 | -.058 | .079 | -.136 | **-.338** |
| O.Unco.2 | R | I know my ideas sometimes surprise people | -.190 | .107 | -.227 | .000 | .023 | **-.328** |
| O.Unco.9 |  | I try to avoid complicated people | -.147 | -.040 | .051 | -.066 | .114 | **.297** |

*Note*. Factor loadings > .30 are in boldface.

NB: In this table, we present the English translation of the Italian items. The original Italian scale can be requested from the authors.

**Table B.**

*Factor loadings (oblimin rotation) of the Principal Component Analysis on the items of the 192-item HEXACO-MSI-E (Study 2).*

| **Item** | **Reversed** | **Stem** | **H** | **E** | **X** | **A** | **C** | **O** |
| --- | --- | --- | --- | --- | --- | --- | --- | --- |
| H.Fair.1 |  | I would never take things that aren't mine | **-.414** | -.069 | .106 | .034 | -.068 | .039 |
| H.Fair.3 |  | If a cashier accidentally gives me extra change, I return it | **-.461** | .016 | .031 | .047 | -.115 | .124 |
| H.Fair.8 | R | If I happen to take advantage of someone, I don't regret it | **.372** | -.140 | -.057 | .003 | -.017 | -.083 |
| H.Fair.9 |  | I wouldn't steal even if I was sure I'd get away with it | **-.554** | .014 | .102 | .008 | -.103 | .002 |
| H.Fair.10 |  | I would never steal something from another guy/girl | **-.535** | -.003 | .091 | .015 | -.051 | -.015 |
| H.Fair.11 | R | I'd like to find a way to get things from the stores without paying | **.438** | -.037 | -.045 | -.045 | .089 | -.031 |
| H.Fair.12 | R | I cheat if I'm sure I won't get caught | **.548** | -.097 | -.070 | -.002 | .149 | -.077 |
| H.Fair.14 | R | I'm curious how I can make a lot of money dishonestly | **.526** | -.127 | -.072 | .111 | .068 | -.103 |
| H.Gree.1 |  | I wouldn't like being a famous celebrity | **-.338** | -.106 | -.159 | .157 | .022 | .035 |
| H.Gree.3 |  | I'm not attracted to luxury | **-.490** | -.034 | -.099 | .159 | .094 | .076 |
| H.Gree.4 |  | I'm not interested in power | **-.527** | .025 | -.028 | .140 | .132 | -.092 |
| H.Gree.11 |  | It's not important to have a lot of money | **-.482** | -.015 | -.041 | .190 | .115 | .041 |
| H.Gree.12 | R | I would like friends who have expensive and fashionable items (mobile, clothes, games) | **.429** | -.097 | -.037 | .096 | -.117 | -.151 |
| H.Gree.13 | R | I really like to have expensive and branded items (mobile phone, clothes, games) | **.460** | .003 | .152 | -.130 | -.107 | -.186 |
| H.Gree.14 | R | I want others to see how important I am | **.494** | .144 | .019 | -.110 | -.100 | .072 |
| H.Gree.16 | R | I want to become famous | **.416** | .097 | .207 | -.119 | -.019 | -.009 |
| H.Mode.1 |  | I don't think I'm better than other people | **-.451** | .033 | -.087 | .143 | .067 | -.070 |
| H.Mode.3 |  | I don't think I'm any better than the others | **-.534** | .028 | -.104 | .096 | .090 | -.059 |
| H.Mode.4 |  | I don't like to attract attention | **-.517** | -.149 | -.260 | .117 | .035 | -.040 |
| H.Mode.6 | R | I'll show off if I get the chance | **.470** | .063 | .285 | -.085 | .013 | .081 |
| H.Mode.7 |  | I'm not the type to brag about my virtues | **-.523** | -.089 | -.035 | .056 | .076 | -.024 |
| H.Mode.10 | R | People have to behave better with me than with the others | **.458** | -.076 | -.047 | -.068 | -.062 | -.093 |
| H.Mode.11 | R | Some say I give myself a lot of airs | **.390** | -.024 | -.022 | -.095 | .047 | .038 |
| H.Mode.16 | R | I'm entitled to special treatment | **.482** | -.019 | -.033 | .089 | -.052 | .039 |
| H.Sinc.1 |  | I don't pretend to be better than I am | **-.366** | -.054 | .093 | .005 | .017 | .050 |
| H.Sinc.4 |  | I'm not good at acting to impress people | **-.427** | .018 | -.021 | -.014 | -.002 | -.243 |
| H.Sinc.10 | R | If I want to get something from people, I try to make friends with them | **.469** | -.092 | -.016 | .015 | .030 | .006 |
| H.Sinc.11 | R | I say nice things to my teachers to get higher grades | **.377** | -.080 | -.007 | .165 | .024 | .009 |
| H.Sinc.13 | R | Sometimes I pretend to be better than I really am | **.416** | .030 | -.166 | -.004 | .086 | .028 |
| H.Sinc.14 | R | Sometimes I lie to get what I want | **.431** | -.026 | -.017 | -.139 | .240 | -.056 |
| H.Sinc.15 |  | I'm not good at pretending with other people | **-.345** | .114 | .196 | .088 | -.026 | -.155 |
| H.Sinc.16 |  | I find it difficult to lie | **-.362** | .132 | .126 | .157 | -.148 | -.059 |
| E.Anxi.2 |  | I worry about everything | -.040 | **.363** | -.171 | -.038 | -.080 | .080 |
| E.Anxi.5 |  | I am easily panicked | -.094 | **.473** | -.238 | -.186 | -.008 | .008 |
| E.Anxi.8 | R | I am not easily upset by what happens around me | -.061 | **-.249** | .083 | .182 | .055 | -.024 |
| E.Anxi.9 | R | I remain calm under pressure | -.010 | **-.370** | .127 | .343 | -.063 | .103 |
| E.Anxi.11 |  | I worry about small things | -.080 | **.405** | -.144 | -.004 | -.122 | .137 |
| E.Anxi.12 | R | I worry less than other people | .172 | **-.359** | -.034 | -.091 | .004 | -.166 |
| E.Anxi.14 |  | I often worry that something may go wrong | -.062 | **.300** | -.125 | -.135 | -.146 | .069 |
| E.Anxi.15 | R | I do not worry as much as other people | .086 | **-.431** | .018 | -.007 | .139 | -.110 |
| E.Depe.1 |  | I need reassurance | .067 | **.450** | -.189 | .015 | .039 | .085 |
| E.Depe.7 |  | I seek support | .071 | **.522** | -.156 | -.003 | .051 | .076 |
| E.Depe.9 |  | When something bad happens, I need someone to cheer me up | .035 | **.634** | .045 | .057 | .048 | .035 |
| E.Depe.11 | R | I can solve difficult situations without encouragement | -.048 | **-.537** | .085 | -.010 | -.045 | .125 |
| E.Depe.13 | R | I can face all my personal problems on my own | .015 | **-.580** | -.060 | -.102 | -.035 | .115 |
| E.Depe.14 | R | I rarely need other people's support | -.017 | **-.499** | -.042 | -.049 | -.075 | -.009 |
| E.Depe.15 |  | I need other people's comfort | .102 | **.578** | -.134 | .036 | .033 | .024 |
| E.Depe.16 | R | I can easily overcome difficulties by myself | .044 | **-.578** | .079 | -.099 | -.054 | .120 |
| E.Fear.8 | R | I play dangerous games fearlessly | .192 | **-.359** | .008 | -.029 | .212 | .145 |
| E.Fear.9 | R | They tell me I am afraid of nothing | .218 | **-.361** | .087 | .044 | .058 | .187 |
| E.Fear.11 |  | If there is a danger, I am very much afraid | -.154 | **.509** | -.010 | -.058 | -.121 | -.143 |
| E.Fear.14 | R | I can easily bear physical pain | .001 | **-.473** | -.019 | .173 | .071 | .161 |
| E.Fear.15 |  | I am afraid of feeling pain | .025 | **.514** | -.034 | -.114 | -.073 | -.096 |
| E.Fear.16 | R | I am braver than other people in dangerous situations | .239 | **-.383** | .078 | -.011 | .130 | .227 |
| E.Fear.17 |  | I'd rather avoid watching scary scenes in a movie | -.075 | **.359** | .031 | -.013 | -.090 | -.154 |
| E.Fear.19 |  | I avoid those sports where it's easy to get hurt | -.111 | **.230** | -.055 | .032 | -.155 | -.167 |
| E.Sent.3 |  | I can cry during a film | .034 | **.444** | -.044 | -.179 | .074 | .173 |
| E.Sent.8 | R | I can’t understand emotional people | .045 | **-.464** | -.224 | .004 | .037 | -.165 |
| E.Sent.9 | R | I am rarely moved | -.039 | **-.507** | -.150 | -.015 | -.082 | -.157 |
| E.Sent.10 |  | If other people cry, I feel like crying too | .034 | **.540** | -.006 | .006 | .045 | .097 |
| E.Sent.13 |  | When somebody is in pain, I feel pain too | .008 | **.427** | -.009 | .159 | .011 | .188 |
| E.Sent.14 |  | Sometimes I feel like crying, when I say someone goodbye | -.001 | **.527** | .066 | -.039 | -.028 | .175 |
| E.Sent.17 | R | People sometimes say I'm insensitive to other people's emotions | .163 | **-.409** | -.227 | -.139 | .101 | -.027 |
| E.Sent.18 | R | I don’t feel like crying, even not in situations where others get emotional | -.083 | **-.641** | -.034 | -.012 | .056 | -.109 |
| X.Socb.4 |  | I have a strong personality | .030 | -.118 | **.282** | -.146 | .011 | .252 |
| X.Socb.5 | R | I'd be afraid to speak in public | -.115 | .243 | **-.434** | .003 | -.057 | -.170 |
| X.Socb.6 | R | I find it difficult to approach others | -.031 | -.042 | **-.598** | -.005 | -.109 | -.112 |
| X.Socb.9 | R | When there are a lot of people I can't say what I think | -.055 | .246 | **-.453** | .160 | -.035 | -.180 |
| X.Socb.13 | R | I stay in the background when I'm in a group | .025 | .065 | **-.617** | .135 | .038 | .020 |
| X.Socb.17 |  | I feel comfortable when I meet new people | .078 | -.118 | **.461** | .174 | .058 | .155 |
| X.Socb.18 |  | When I am in a group I can easily express what I think | .036 | -.125 | **.636** | -.049 | .036 | .086 |
| X.Socb.19 |  | I am not embarrassed to speak in front of an audience | .053 | -.272 | **.438** | .016 | .066 | .180 |
| X.Soci.1 |  | I usually like to spend my free time with people | -.016 | .192 | **.559** | .098 | .020 | -.040 |
| X.Soci.3 |  | I make friends easily | .028 | .032 | **.627** | .127 | .150 | .061 |
| X.Soci.5 | R | I seem to get less pleasure than others from interacting with people | .094 | -.077 | **-.515** | .046 | -.001 | -.004 |
| X.Soci.6 | R | I rarely enjoy being with people | .054 | -.194 | **-.461** | -.009 | -.074 | -.061 |
| X.Soci.7 | R | I keep others at a distance | .064 | -.250 | **-.529** | -.100 | -.032 | .040 |
| X.Soci.10 |  | It's nice to have a lot of friends to talk to | -.140 | .255 | **.535** | .043 | -.013 | -.099 |
| X.Soci.12 |  | When I'm in a new place, I try to make friends immediately | .069 | .119 | **.573** | .134 | .121 | .112 |
| X.Soci.14 | R | I prefer to be alone | -.038 | -.189 | **-.604** | -.149 | -.005 | .090 |
| X.Live.3 |  | I smile so much | .027 | .118 | **.559** | .169 | .047 | .032 |
| X.Live.6 | R | I don't think I have much inner strength | -.061 | .340 | **-.367** | .063 | .076 | -.108 |
| X.Live.7 |  | I have so much fun | .036 | -.022 | **.478** | .061 | .009 | .067 |
| X.Live.9 | R | I often feel down | .093 | .232 | **-.489** | -.094 | .097 | .116 |
| X.Live.10 |  | I'm usually cheerful and positive | -.039 | .039 | **.647** | .175 | -.059 | -.027 |
| X.Live.13 | R | Most of my friends are more optimistic and active than I am | -.014 | .042 | **-.514** | -.002 | .120 | -.012 |
| X.Live.14 | R | I'm often in a gloomy mood | .060 | .059 | **-.582** | -.139 | .078 | .069 |
| X.Live.15 |  | I'm usually cheerful | .010 | .031 | **.651** | .154 | -.081 | -.099 |
| X.SSes.2 | R | I don't think the others like me very much | -.032 | .180 | **-.552** | -.053 | .076 | .067 |
| X.SSes.3 | R | I feel unimportant | -.062 | .168 | **-.586** | .033 | .126 | .045 |
| X.SSes.4 |  | People like me | -.026 | -.061 | **.596** | -.013 | -.047 | -.014 |
| X.SSes.6 | R | Nobody likes talking to me | -.002 | -.035 | **-.633** | .084 | .037 | .066 |
| X.SSes.8 | R | I don't have much self-esteem | -.076 | .155 | **-.552** | -.019 | .136 | .004 |
| X.SSes.9 |  | I hardly ever feel ignored | -.081 | -.212 | **.538** | .070 | -.039 | -.129 |
| X.SSes.10 |  | I'm pleased with myself | -.004 | -.176 | **.593** | .013 | -.180 | -.105 |
| X.SSes.13 |  | Others enjoy spending time with me | -.091 | -.035 | **.617** | -.052 | -.058 | -.002 |
| A.Flex.2 |  | I am good at taking advice | -.136 | .078 | .236 | **.276** | -.121 | .065 |
| A.Flex.5 | R | I hate to be contradicted | .139 | .075 | .017 | **-.528** | -.047 | -.052 |
| A.Flex.10 | R | My parents say I always want to be right | .120 | .038 | -.012 | **-.416** | .097 | .044 |
| A.Flex.11 | R | When people tell me I'm wrong, I get in arguments with them | .241 | -.006 | -.092 | **-.515** | .046 | -.051 |
| A.Flex.15 | R | Others struggle to change my mind | -.008 | -.077 | .048 | **-.510** | .081 | .038 |
| A.Flex.17 |  | I like asking others for advice to see if I'm doing right | -.011 | **.326** | .141 | **.253** | .013 | -.028 |
| A.Flex.19 |  | To avoid conflict, I adapt to what others say even if I don't agree with it | .028 | .083 | -.136 | **.384** | .029 | -.111 |
| A.Flex.20 |  | I have no problem changing my opinion if it is explained to me why | -.147 | .078 | .130 | **.212** | .009 | -.106 |
| A.Forg.2 |  | I'm prone to forgiving others | -.164 | .152 | .127 | **.466** | .042 | .033 |
| A.Forg.4 | R | I find it hard to forgive others | .053 | -.150 | -.105 | **-.515** | -.077 | -.039 |
| A.Forg.10 |  | If someone makes me angry, I usually get over it soon | -.122 | .003 | .139 | **.528** | .006 | .012 |
| A.Forg.11 |  | If people treat me badly, I forgive them and forget about it | -.061 | .064 | .015 | **.564** | .086 | .063 |
| A.Forg.12 | R | If someone misbehaves with me, they can't be my friend anymore | .129 | -.116 | -.058 | **-.428** | -.123 | -.073 |
| A.Forg.13 | R | I remain unfriendly to those who have been bad to me | .069 | -.021 | -.066 | **-.505** | -.114 | -.114 |
| A.Forg.14 |  | I'm quick to restore confidence to those who tricked and cheated me | -.008 | .103 | .054 | **.484** | .146 | .019 |
| A.Forg.15 | R | I'm wary of people who have wronged me | -.021 | -.141 | -.083 | **-.429** | -.021 | -.057 |
| A.Gent.5 | R | I judge others very quickly | .243 | -.020 | .032 | **-.283** | .053 | -.114 |
| A.Gent.6 | R | I find flaws in everything | .188 | -.011 | -.185 | **-.386** | -.041 | -.102 |
| A.Gent.9 |  | I'm a nice, quiet guy/girl | -.226 | .117 | .167 | **.292** | **-.282** | -.021 |
| A.Gent.12 |  | Even when people make many mistakes, I don't judge them negatively | **-.419** | -.005 | .033 | **.320** | .049 | .076 |
| A.Gent.13 | R | I often express criticism | .199 | -.048 | .060 | **-.357** | .104 | -.010 |
| A.Gent.15 | R | I react negatively to people who make mistakes | **.306** | -.001 | -.102 | **-.288** | -.042 | -.037 |
| A.Gent.18 |  | It comes naturally to me to be kind and courteous to others | -.198 | .186 | .248 | **.285** | -.167 | .048 |
| A.Gent.19 |  | I don't like to judge others harshly | **-.441** | .037 | .073 | .183 | .041 | .011 |
| A.Pati.1 |  | It takes a lot to make me angry at someone | -.037 | -.008 | .083 | **.520** | -.032 | .038 |
| A.Pati.3 |  | I'm usually a patient person | -.073 | -.021 | .016 | **.513** | -.205 | .033 |
| A.Pati.5 |  | I rarely get angry | -.040 | -.048 | .092 | **.546** | -.112 | -.069 |
| A.Pati.6 | R | I'm easily irritable | .018 | .001 | -.219 | **-.528** | .120 | -.018 |
| A.Pati.7 | R | I get angry easily | -.033 | .021 | -.088 | **-.643** | .142 | -.073 |
| A.Pati.9 | R | I lose my patience | .010 | .042 | -.041 | **-.593** | .177 | -.091 |
| A.Pati.11 | R | My parents think I get angry easily | .025 | .032 | -.078 | **-.526** | .147 | -.019 |
| A.Pati.15 |  | Even when I'm treated badly, I stay calm | -.029 | -.148 | -.045 | **.589** | .004 | .085 |
| C.Dili.2 |  | I'm very demanding when I do my homework | -.165 | .042 | .044 | -.115 | **-.455** | .141 |
| C.Dili.3 |  | I work hard | -.191 | -.061 | .147 | -.065 | **-.456** | .211 |
| C.Dili.4 |  | I finish my tasks successfully | -.203 | -.104 | .165 | -.126 | **-.464** | .172 |
| C.Dili.5 | R | I only do my tasks to get by | .275 | -.082 | -.094 | .145 | **.358** | -.132 |
| C.Dili.7 |  | I study a lot | -.261 | .037 | .069 | -.174 | **-.535** | .200 |
| C.Dili.15 | R | I postpone as long as possible the tasks that seem difficult to me | .162 | -.024 | -.104 | .013 | **.324** | -.132 |
| C.Dili.17 | R | If a task is difficult I tend to give up | .133 | .126 | -.182 | .032 | **.251** | -.207 |
| C.Dili.19 | R | When I have a task to do, I do the bare minimum | .219 | .037 | -.063 | .190 | **.346** | -.150 |
| C.Orga.1 |  | I keep things in order | .094 | .008 | .013 | .119 | **-.679** | -.092 |
| C.Orga.3 |  | I like to tidy up | .139 | .061 | -.093 | .124 | **-.604** | .004 |
| C.Orga.5 | R | My room is often messy | -.189 | -.009 | .000 | -.131 | **.618** | .112 |
| C.Orga.6 | R | I leave my things lying around | -.148 | .066 | .089 | -.215 | **.627** | .071 |
| C.Orga.8 | R | I often forget to put things back where they belong | -.204 | .037 | .053 | -.154 | **.668** | .045 |
| C.Orga.10 |  | I like to keep things organized | .131 | .055 | -.023 | .021 | **-.680** | -.003 |
| C.Orga.11 |  | To avoid confusion, I decide what I have to do first | -.066 | -.015 | .004 | .064 | **-.463** | .027 |
| C.Orga.15 | R | I have trouble finding things because I'm messy | -.166 | .046 | .066 | -.182 | **.695** | .089 |
| C.Perf.9 | R | I'm not very precise when I do my homework | .102 | -.019 | -.108 | .178 | **.580** | -.167 |
| C.Perf.10 |  | I always try to be precise in my homework, even if it takes me longer | -.190 | .007 | .066 | -.075 | **-.560** | .104 |
| C.Perf.11 |  | People consider me very precise | -.001 | .062 | .095 | -.090 | **-.569** | .065 |
| C.Perf.13 |  | I usually check carefully how I did my homework | -.139 | .029 | .075 | -.072 | **-.545** | .192 |
| C.Perf.14 | R | I think it's a waste of time checking my homework for mistakes | .235 | -.128 | -.079 | .109 | **.346** | -.201 |
| C.Perf.15 |  | I'm very precise when I do my homework | -.194 | -.019 | .076 | -.141 | **-.662** | .119 |
| C.Perf.18 | R | I don't like to waste time perfecting a job if it's already fine as it is | .048 | -.034 | -.014 | .056 | **.392** | -.240 |
| C.Perf.19 | R | When I finish doing something (e.g., homework) I don't recheck if there are any mistakes that I missed | .107 | -.027 | -.065 | .045 | **.425** | -.229 |
| C.Prud.1 |  | I make plans and I stick to them | -.006 | .016 | .095 | .004 | **-.258** | **.267** |
| C.Prud.2 |  | I do things according to a plan | .087 | -.056 | -.014 | .073 | **-.381** | .101 |
| C.Prud.3 | R | I dive into things without thinking | .128 | -.042 | -.006 | -.104 | **.440** | .007 |
| C.Prud.4 |  | When I do something I always think about the consequences | -.125 | .101 | .002 | .157 | **-.303** | .126 |
| C.Prud.5 | R | I make rash decisions | .086 | .000 | -.006 | -.162 | **.399** | -.143 |
| C.Prud.11 | R | I'm often wrong because I do things without thinking | .090 | .088 | -.097 | -.038 | **.460** | -.163 |
| C.Prud.13 |  | I think carefully before I do something dangerous | -.211 | .230 | .077 | .088 | **-.351** | -.018 |
| C.Prud.16 | R | I often do things without really thinking about it | .107 | -.038 | -.011 | -.121 | **.474** | -.092 |
| O.Aesa.1 |  | I believe in the importance of art | -.125 | .029 | -.070 | .044 | -.059 | **.551** |
| O.Aesa.5 |  | I've read some classics of literature | -.024 | -.042 | -.131 | .018 | -.124 | **.436** |
| O.Aesa.6 | R | I don't like art | .003 | -.063 | .116 | -.042 | .027 | **-.531** |
| O.Aesa.10 | R | I get bored visiting a museum | .084 | -.023 | .070 | -.099 | .083 | **-.535** |
| O.Aesa.11 | R | Reading poetry is a waste of time | .131 | -.128 | .058 | -.106 | .209 | **-.440** |
| O.Aesa.13 |  | I could be watching a painting for a long time | -.018 | -.015 | -.250 | .151 | -.082 | **.612** |
| O.Aesa.14 | R | I think most art is boring | .071 | -.081 | .142 | -.115 | .017 | **-.583** |
| O.Aesa.15 |  | I love poetry | -.039 | .018 | -.063 | .157 | -.200 | **.516** |
| O.Crea.1 | R | I'm not a guy/girl with original thoughts and ideas | -.112 | -.038 | -.141 | .154 | .034 | **-.340** |
| O.Crea.3 | R | I don't have many ideas | -.078 | .010 | -.207 | .089 | -.029 | **-.516** |
| O.Crea.6 | R | I don't have a good imagination | -.086 | -.028 | -.213 | .093 | -.030 | **-.474** |
| O.Crea.10 |  | I'd like to create a work of art, such as a story, a song or a painting | -.018 | .038 | -.103 | .034 | .026 | **.606** |
| O.Crea.11 |  | People tell me I have a lot of imagination | .105 | -.026 | .255 | -.031 | .098 | **.515** |
| O.Crea.12 | R | I'll never become an artist | -.131 | -.050 | -.023 | -.046 | -.024 | **-.452** |
| O.Crea.13 |  | I have a lot of imagination | .039 | .034 | .216 | -.008 | .084 | **.512** |
| O.Crea.14 |  | I like to think of new ways of doing things | .011 | .060 | .186 | -.063 | -.012 | **.341** |
| O.Inqu.1 |  | I'm interested in science | -.152 | -.055 | .024 | .038 | -.173 | **.415** |
| O.Inqu.7 | R | I avoid reading things that are too complicated | .092 | .116 | .060 | .005 | .178 | **-.410** |
| O.Inqu.8 | R | I'm not worried about political and social problems | -.093 | -.111 | .064 | -.007 | .109 | **-.397** |
| O.Inqu.10 |  | I like to know what happens in other countries | .010 | .006 | -.002 | .116 | -.111 | **.393** |
| O.Inqu.11 | R | Documentaries about nature are boring | .060 | -.045 | .091 | -.194 | .066 | **-.448** |
| O.Inqu.14 |  | I like to read about new scientific discoveries | -.050 | -.100 | -.052 | .094 | -.147 | **.509** |
| O.Inqu.15 | R | I think science is boring | .068 | -.030 | .028 | -.072 | .146 | **-.465** |
| O.Inqu.16 |  | I'd like to read a book about nature | -.067 | -.011 | -.078 | .187 | -.160 | **.488** |
| O.Unco.16 |  | People are surprised by my opinions | .167 | -.132 | .102 | -.059 | .069 | **.372** |
| O.Unco.17 |  | I often behave in a more original way than others | .284 | -.101 | .067 | -.108 | -.047 | **.300** |
| O.Unco.19 |  | I like to be considered an original/out-of-the-ordinary guy | .266 | .062 | .058 | -.134 | .053 | **.334** |
| O.Unco.20 |  | People are sometimes amazed at the originality of my ideas | .171 | -.059 | .266 | -.072 | .013 | **.462** |
| O.Unco.21 | R | I prefer to hang out with guys or girls who think like everyone else | .081 | -.057 | .185 | .189 | -.013 | **-.253** |
| O.Unco.22 | R | There is nothing wrong with behaving like others | -.020 | -.049 | .058 | **.188** | .053 | -.166 |
| O.Unco.23 | R | I feel more comfortable hanging out with guys/girls who act like everyone else | .072 | -.026 | .219 | .197 | .002 | **-.222** |
| O.Unco.24 | R | I'm not usually impressed with original ideas | -.009 | -.139 | -.091 | .165 | .092 | **-.379** |

NB: In this table, we present the English translation of the Italian items. The original Italian scale can be requested from the authors.

**Table C.**

*Correlations among the six factors extracted from the facets (CFA) of the 192-item HEXACO-MSI (Study 2).*

| Factor | H | E | X | A | C |
| --- | --- | --- | --- | --- | --- |
| H |  |  |  |  |  |
| E | .304 |  |  |  |  |
| X | .126 | -.435 |  |  |  |
| A | .589 | .097 | .368 |  |  |
| C | .496 | .095 | .314 | .363 |  |
| O | .350 | .147 | .153 | .322 | .665 |

*Note.*

H = Honesty/Humility; E = Emotionality; X = Extraversion; A = Agreeableness; C = Conscientiousness; O = Openness to Experience.

**Table D.**

*Correlations among the six factors of the final version of the 192-item HEXACO-MSI-E based on scale score (Study 2).*

| Factor | H | E | X | A | C |
| --- | --- | --- | --- | --- | --- |
| H |  |  |  |  |  |
| E | .283** |  |  |  |  |
| X | .099** | -.179** |  |  |  |
| A | .556** | .175** | .341** |  |  |
| C | .371** | .126** | .305** | .385** |  |
| O | .194** | .100** | .200** | .203** | .517** |

*Note.*

H = Honesty/Humility; E = Emotionality; X = Extraversion; A = Agreeableness; C = Conscientiousness; O = Openness to Experience.

**Table E.**

*Paired samples T-test with FDR correction for the six traits of personality at times t_1_ and t_2_. (N = 182, df = 181).*

| Factor | Mean t_1_ | SD | Mean t_2_ | SD | t | p | d |
| --- | --- | --- | --- | --- | --- | --- | --- |
| H | 3.86 | .59 | 3.84 | .57 | .59 | .557 | .04 |
| E | 3.38 | .53 | 3.33 | .58 | 1.47 | .143 | .11 |
| X | 3.80 | .65 | 3.59 | .73 | 5.32 | .000 * | .39 |
| A | 3.15 | .67 | 3.23 | .71 | -2.07 | .040 | -.15 |
| C | 3.40 | .76 | 3.36 | .82 | .96 | .338 | .07 |
| O | 3.39 | .65 | 3.36 | .66 | .86 | .393 | .06 |

* Only values of p < .0083 are significant with FDR correction for multiple testing.

**Table F.**

*Paired samples T-test with FDR correction for the 24 facets of personality at times t_1_ and t_2_ (N = 182, DF = 181).*

| Facet | Mean t_1_ | SD | Mean t_2_ | SD | t | p | d |
| --- | --- | --- | --- | --- | --- | --- | --- |
| Sincerity | 3.86 | .73 | 3.85 | .74 | .16 | .876 | .01 |
| Fairness | 4.26 | .71 | 4.25 | .70 | .08 | .938 | .01 |
| Greed Avoidance | 3.31 | .76 | 3.27 | .78 | .89 | .374 | .07 |
| Modesty | 4.00 | .69 | 3.97 | .68 | .57 | .568 | .04 |
| Fearfulness | 3.12 | .84 | 3.02 | .77 | 1.95 | .053 | .14 |
| Anxiety | 3.52 | .67 | 3.58 | .67 | -1.34 | .181 | -.10 |
| Dependence | 3.15 | .82 | 3.13 | .87 | .32 | .752 | .02 |
| Sentimentality | 3.72 | .76 | 3.57 | .95 | 2.58 | .011 * | .19 |
| Social Self-Esteem | 3.75 | .91 | 3.46 | .93 | 4.87 | .000 * | .36 |
| Social Boldness | 3.38 | .78 | 3.32 | .84 | .92 | .357 | .07 |
| Sociability | 4.11 | .70 | 3.96 | .80 | 3.17 | .002 | .24 |
| Liveliness | 3.98 | .75 | 3.59 | .84 | 7.47 | .000 * | .55 |
| Forgivingness | 3.23 | .84 | 3.08 | .94 | 2.50 | .013 * | .19 |
| Gentleness | 3.43 | .62 | 3.75 | .72 | -7.10 | .000 * | -.53 |
| Flexibility | 2.95 | .79 | 3.18 | .73 | -4.25 | .000 * | -.31 |
| Patience | 2.98 | 1.00 | 2.89 | 1.04 | 1.32 | .187 | .10 |
| Organization | 3.22 | 1.04 | 3.19 | 1.11 | .42 | .677 | .03 |
| Diligence | 3.56 | .82 | 3.57 | .96 | -.35 | .730 | -.03 |
| Perfectionism | 3.58 | .93 | 3.36 | 1.02 | 4.01 | .000 * | .30 |
| Prudence | 3.22 | .83 | 3.30 | .87 | -1.28 | .204 | -.09 |
| Aesthetic Appreciation | 3.24 | .93 | 3.14 | .99 | 1.83 | .070 | .14 |
| Inquisitiveness | 3.41 | .90 | 3.29 | .93 | 2.10 | .037 | .16 |
| Creativity | 3.68 | .80 | 3.56 | .83 | 2.25 | .026 | .17 |
| Unconventionality | 3.24 | .65 | 3.46 | .70 | -3.71 | .000 * | -.27 |

* Only values of p < .01875 are significant with FDR correction for multiple testing.
